# Cancer-associated fibroblast-related prognostic signature predicts prognosis and immunotherapy response in pancreatic adenocarcinoma based on single-cell and bulk RNA-sequencing

**DOI:** 10.1038/s41598-023-43495-y

**Published:** 2023-09-29

**Authors:** Yajun Chen, Qican Deng, Hui Chen, Jianguo Yang, Zhenzhou Chen, Juncai Li, Zhongxue Fu

**Affiliations:** 1 Department of General Surgery, The Third Affiliated Hospital of Chongqing Medical University, Chongqing, China; 2 Institute of Hepatopancreatobiliary Surgery, Chongqing General Hospital, Chongqing, China; 3 Department of Gastrointestinal Surgery, The First Affiliated Hospital of Chongqing Medical University, Chongqing, China; 4 Department of Surgery, The People’s Hospital of Yubei District of Chongqing, Chongqing, China

**Keywords:** Computational biology and bioinformatics, Data acquisition, Data integration, Data mining, Predictive medicine, Tumour biomarkers, Tumour immunology, Pancreatic cancer

## Abstract

Cancer-associated fibroblasts (CAFs) influence many aspects of pancreatic adenocarcinoma (PAAD) carcinogenesis, including tumor cell proliferation, angiogenesis, invasion, and metastasis. A six-gene prognostic signature was constructed for PAAD based on the 189 CAF marker genes identified in single-cell RNA-sequencing data. Multivariate analyses showed that the risk score was independently prognostic for survival in the TCGA (P < 0.001) and ICGC (P = 0.004) cohorts. Tumor infiltration of CD8 T (P = 0.005) cells and naïve B cells (P = 0.001) was greater in the low-risk than in the high-risk group, with infiltration of these cells negatively correlated with risk score. Moreover, the TMB score was lower in the low-risk than in the high-risk group (P = 0.0051). Importantly, patients in low-risk group had better immunotherapy responses than in the high-risk group in an independent immunotherapy cohort (IMvigor210) (P = 0.039). The CAV1 and SOD3 were highly expressed in CAFs of PAAD tissues, which revealed by immunohistochemical staining. In summary, this comprehensive analysis resulted in the development of a novel prognostic signature, which was associated with immune cell infiltration, drug sensitivity, and TMB, and could predict the prognosis and immunotherapy response of patients with PAAD.

## Introduction

Pancreatic adenocarcinoma (PAAD) is a highly lethal tumor due to its relatively late diagnosis, rapid metastatic dissemination, and limited methods of treatment^1^. The American Cancer Society has estimated that, in 2022, 62,210 individuals would develop pancreatic cancer, that 49,830 would die of this disease, and that the 5-year relative survival rate would be 11%^2^. Because PAAD is often diagnosed in later stages, surgical resection is feasible only in a small percentage of these patients, with the 5-year survival rate after surgery being only 20%^3^. Moreover, although immunotherapeutic strategies, including treatment with immune-checkpoint inhibitors, have shown promise in many other malignancies, these strategies have had limited effects in patient with PAAD^4,5^. Thus, it is necessary to identify novel potential treatment targets, based on factors prognostic of survival, in patients with PAAD.

Poor patient response to immunotherapeutic strategies may be due to the complicated and highly heterogeneous pancreatic tumor microenvironment (TME), consisting of cancer cells, cancer-associated fibroblasts (CAFs), infiltrating immune cells, components of the extracellular matrix (ECM), and other signaling molecules^1,6^. CAFs within the TME maintain the ECM and play significant roles in the malignant progression of PAAD^7,8^. Specifically, CAFs promote desmoplastic stroma by secreting ECM proteins, thus forming a physical and metabolic barrier that reduces the effects of various immunotherapeutic agents^7,9–11^. Moreover, CAFs secrete many cytokines and chemokines, such as transforming growth factor-β (TGF-β), CXC chemokine ligand 12 (CXCL12), fibroblast growth factor 5 (FGF5), vascular endothelial growth factor (VEGF), growth differentiation factor 15 (GDF15), and interleukin-6 (IL-6), which establish an immunosuppressive environment and modulate the invasion and proliferation of PAAD cells^12–14^. CAFs can also affect tumor related immunosuppression, metabolic reprogramming, angiogenesis induction, tissue invasion, and metastasis by interacting with various infiltrating immune cells in the TME^15,16^.

Many recent bioinformatics studies have explored the relationship between CAF-related genes and tumor prognosis. Prognostic models consisting of CAF-related gene signatures were constructed to explore the prognosis and response to immunotherapy of patients with gastric cancer, colon cancer, and hepatocellular carcinoma^17–19^. The present study describes the construction of a CAF marker gene signature predictive of prognosis in patients with PAAD. These showed that CAF marker genes may be possible prognostic markers and therapeutic targets in PAAD.

## Results

### Identification of CAF marker genes

**Figure 1:**
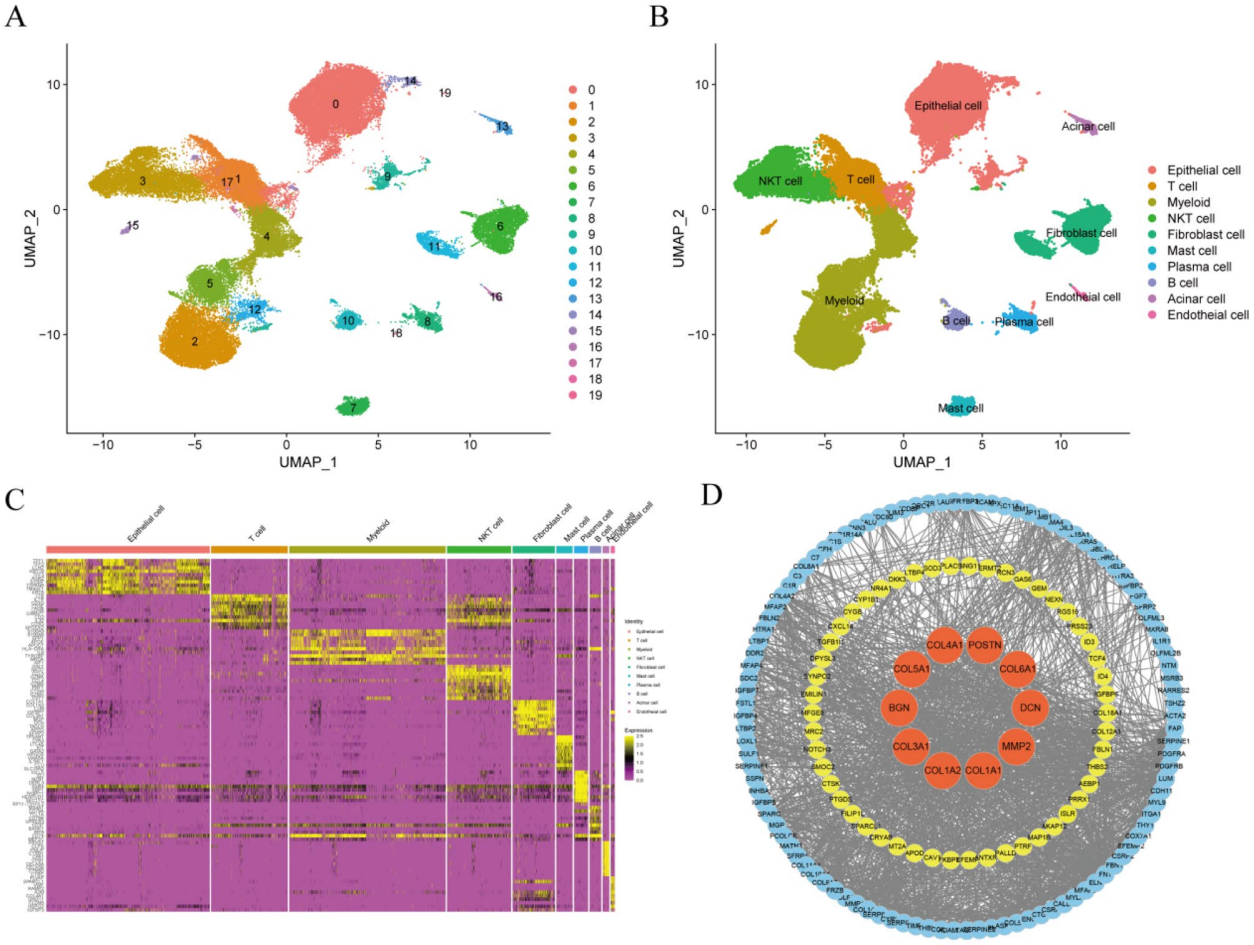
Identification of CAF cell marker genes by single-cell RNA-sequencing analysis. (**A**) UMAP plot of 57,486 cells from 30 primary PAAD samples. (**B**) UMAP plot colored by 10 cell clusters. (**C**) Heatmap identification of the top 10 marker genes in each cell cluster. (**D**) STRING database identification of the PPI network formed by 189 CAF marker genes (interaction score = 0.4).

A total of 57,486 cells of 30 primary PAAD samples were collected and identified as 20 distinct cell clusters (Fig. 1A). In addition, 10 cell clusters were identified based on cell markers from previously described cell markers^20^ and on CellMarker (Fig. 1B). DEGs in each cluster were identified using the “FindAllMarkers” function, with the top 10 DEGs in each cluster determined via the “DoHeatmap” function (Fig. 1C). The heatmap demonstrated that the highly expressed genes in each cluster were predominantly specific to that particular cluster, indicating the reliability of the cell cluster identification. Subsequently, 189 DEGs exhibiting |Log2FC| > 1 and a P-value < 0.05 between the fibroblast cluster and other clusters were identified as CAF marker genes. Their protein–protein interaction (PPI) network was visualized using Search Tool for the Retrieval of Interacting Genes (STRING) database and Cytoscape, and hub genes were identified using the MCC method. Among the top ten hub genes identified were COLA1A, COL1A2, COL3A1, BGN, COL5A1, COL4A1, POSTN, COL6A1, DCN, and MMP2 (Fig. 1D).

### Establishment of the six-gene prognostic signature based on CAF marker genes

**Figure 2:**
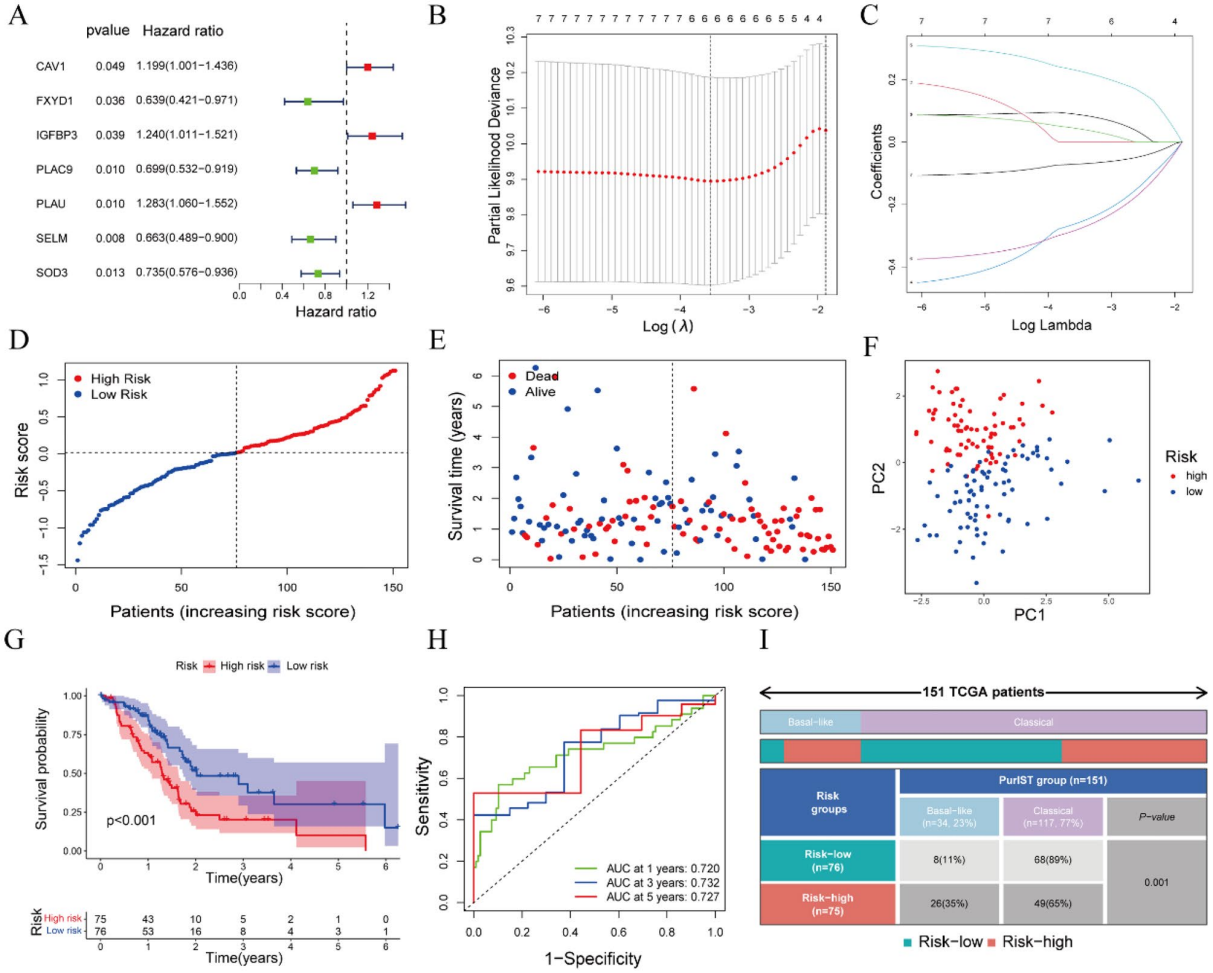
Establishment of a six-gene prognostic signature based on CAF marker genes. (**A**) Forest map showing the seven genes with P < 0.05 obtained by univariate Cox regression analysis. (**B**) LASSO regression of the seven OS-related genes. (**C**) Tuning parameter (λ) selection cross-validation curve of these seven genes. (**D**) Division of PAAD patients into high-risk and low-risk groups based on median risk score. (**E**) Survival status of patients in the two subgroups (Blue dot: Alive, Red dot: Dead). (**F**) PCA plots according to risk scores in PAAD patients. (**G**) Kaplan–Meier analysis of OS of PAAD patients in the two subgroups. (**H**) ROC curve analysis of the prognostic efficiency of the risk model. (**I**) The relationship between CAF-associated prognostic subtypes (low-risk and high-risk group) and PDAC tumor subtypes (basal-like and classical group) in TCGA (P = 0.001).

Univariate Cox regression analysis of the relationships between these 189 DEGs and the survival of patients with PAAD was performed to identify survival-related genes. Based on a criterion of P < 0.05, seven genes, *CAV1*, *FXYD1*, *IGFBP3*, *PLAC9*, *PLAU*, *SELM*, and *SOD3*, were found to associated with patient survival (Fig. 2A) and further analyzed. Three of these genes (*CAV1*, *IGFBP3*, and *PLAU*) with HRs > 1 were related to increased risk, whereas the other four genes (*FXYD1*, *PLAC9*, *SELM*, and *SOD3*) with HRs < 1 were considered protective genes. These seven genes were subjected to LASSO-Cox regression analysis with tenfold cross-validation. Six of these genes (*CAV1*, *IGFBP3*, *PLAC9*, *PLAU*, *SELM*, and *SOD3*) were utilized to construct a prognostic signature based on the optimum λ value (Fig. 2B,C).

The risk score for each patient with PAAD was calculated using the formula: Risk score = (0.088 × *CAV1* exp.) + (0.044 × *IGFBP3* exp.) + (− 0.261 × *PLAC9* exp.) + (0.229 × *PLAU* exp.) + (− 0.282 × *SELM* exp.) + (− 0.071 × *SOD3* exp.). Based on the median risk score, the 151 patients with PAAD were divided into low-risk and high-risk groups (Fig. 2D). Survival time was longer and survival rate higher in the low-risk than in the high-risk group (Fig. 2E). At the same time, the patients in low-risk and high-risk groups were effectively distributed into two directions based on principal component analysis (PCA) (Fig. 2F). Subsequent assessment of the prognostic value of risk score using the Kaplan–Meier method showed that the probability of survival was significantly higher and (overall survival) OS time significantly longer in the low-risk than in the high-risk group (P < 0.001, Fig. 2G). The sensitivity and specificity of the prognostic risk model was evaluated by time-dependent ROC analysis, which found that the AUCs at 1 year, 3 years, and 5 years were 0.720, 0.732, and 0.727, respectively, indicating that this risk model was both accurate and sensitive in predicting the prognosis of patients with PAAD (Fig. 2H). Subsequently, we conducted further investigations into the relationship between low-risk and high-risk group and PAAD tumor subtypes (basal-like and classical group), which were identified utilizing a Purity Independent Subtyping of Tumors (PurIST) classifier^21^. Our findings revealed that a significant majority of patients in the low-risk group were also classified into the classical group (89%). Moreover, both the low-risk group and classical group exhibited substantially longer OS compared to the low-risk group and basal-like group (Fig. 2I). Additionally, statistical analysis demonstrated significant distribution differences between the low-risk and high-risk groups across the basal-like and classical groups (P = 0.001, Fig. 2I).

### External validation of the prognostic signature

**Figure 3:**
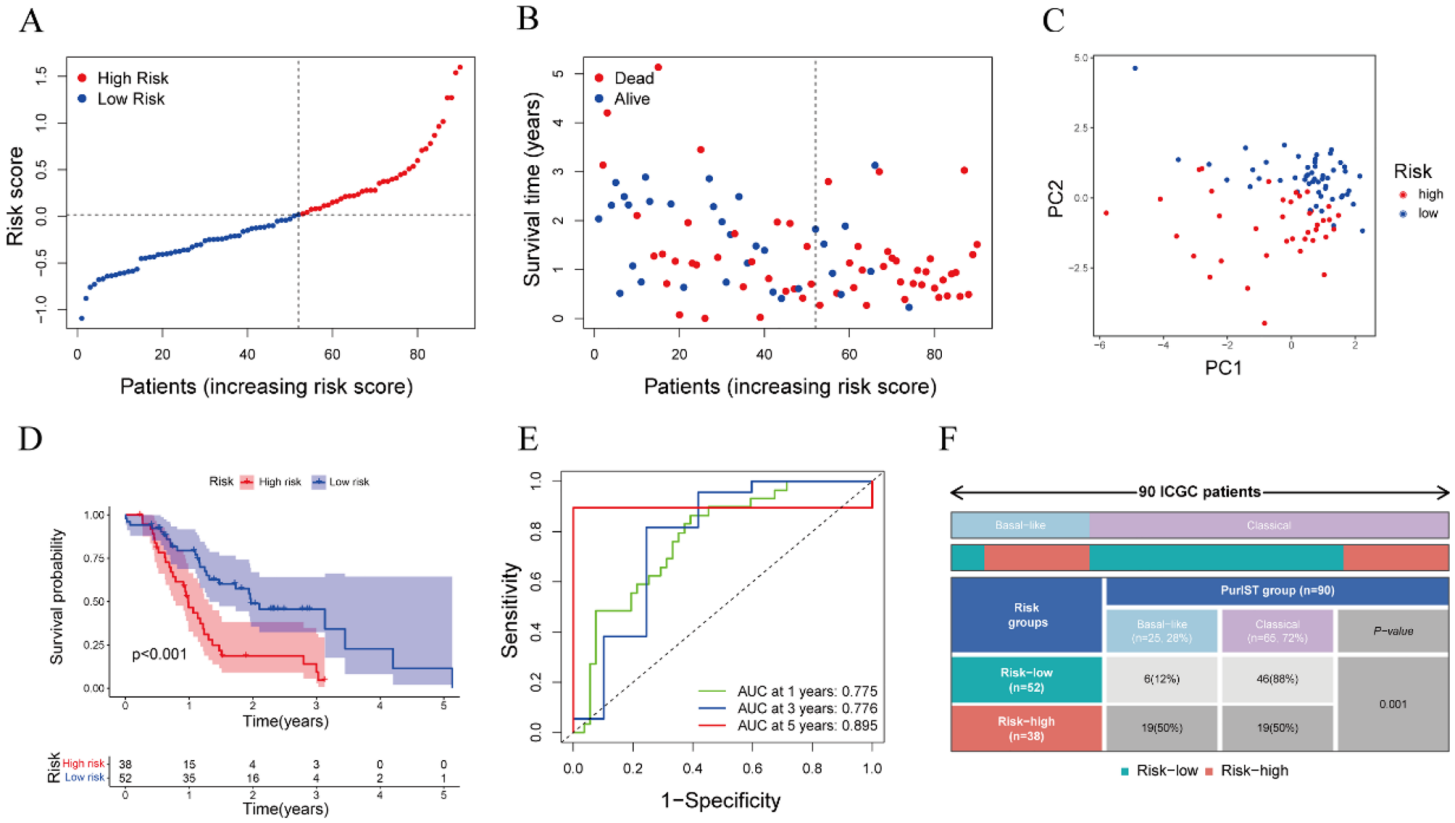
Validation of the CAF marker gene prognostic signature in the ICGC dataset. (**A**) Division of PAAD patients in the ICGC dataset into high- and low-risk groups based on the median risk score in the TCGA cohort. (**B**) Distribution of survival status of high- and low-risk PAAD patients in the ICGC dataset (Blue dot: Alive, Red dot: Dead). (**C**) PCA plot based on the risk scores of PAAD patients in the ICGC dataset. (**D**) Kaplan–Meier analysis of OS of PAAD patients in the low- and high-risk subgroups, with comparisons by log-rank tests. (**E**) ROC curves for 1, 3, and 5 year overall survival of high- and low-risk PAAD patients in the ICGC dataset. (**F**) The relationship between CAF-associated prognostic subtypes (low-risk and high-risk group) and PDAC tumor subtypes (basal-like and classical group) in ICGC (P = 0.001).

The robustness of the CAF marker gene prognosis signature was validated in the PACA-AU dataset, which included RNA-seq data and clinical information on 90 patients with PAAD. Patient risk scores were calculated as described above, and patients were sorted into low-risk and high-risk groups according to the median risk score of the TCGA dataset (Fig. 3A). Similar to results in the TCGA cohort, survival rate was higher in the low-risk than in the high-risk group (Fig. 3B). Meanwhile, the patients in low-risk and high-risk groups of ICGC dataset were also distributed into two directions by using PCA (Fig. 3C). Then, the result that patients with low-risk scores had remarkably longer OS than patients with high-risk scores was revealed by Kaplan–Meier analysis (Fig. 3D). The sensitivity and specificity of the prognostic risk model were validated by time-dependent ROC analysis, which showed that the AUCs for 1-, 3-, and 5-year survival were 0.775, 0.776, and 0.895, respectively (Fig. 3E). In a similar vein, we observed that a majority of patients in the low-risk group were also classified into the classical group (88%). Furthermore, significant distribution differences between the low-risk and high-risk groups were evident across the basal-like and classical groups (P = 0.001, Fig. 3F). Taken together, these results demonstrated the robustness of the CAF marker gene prognosis signature.

### Evaluation of the independent prognostic value of risk score and clinical features

**Figure 4:**
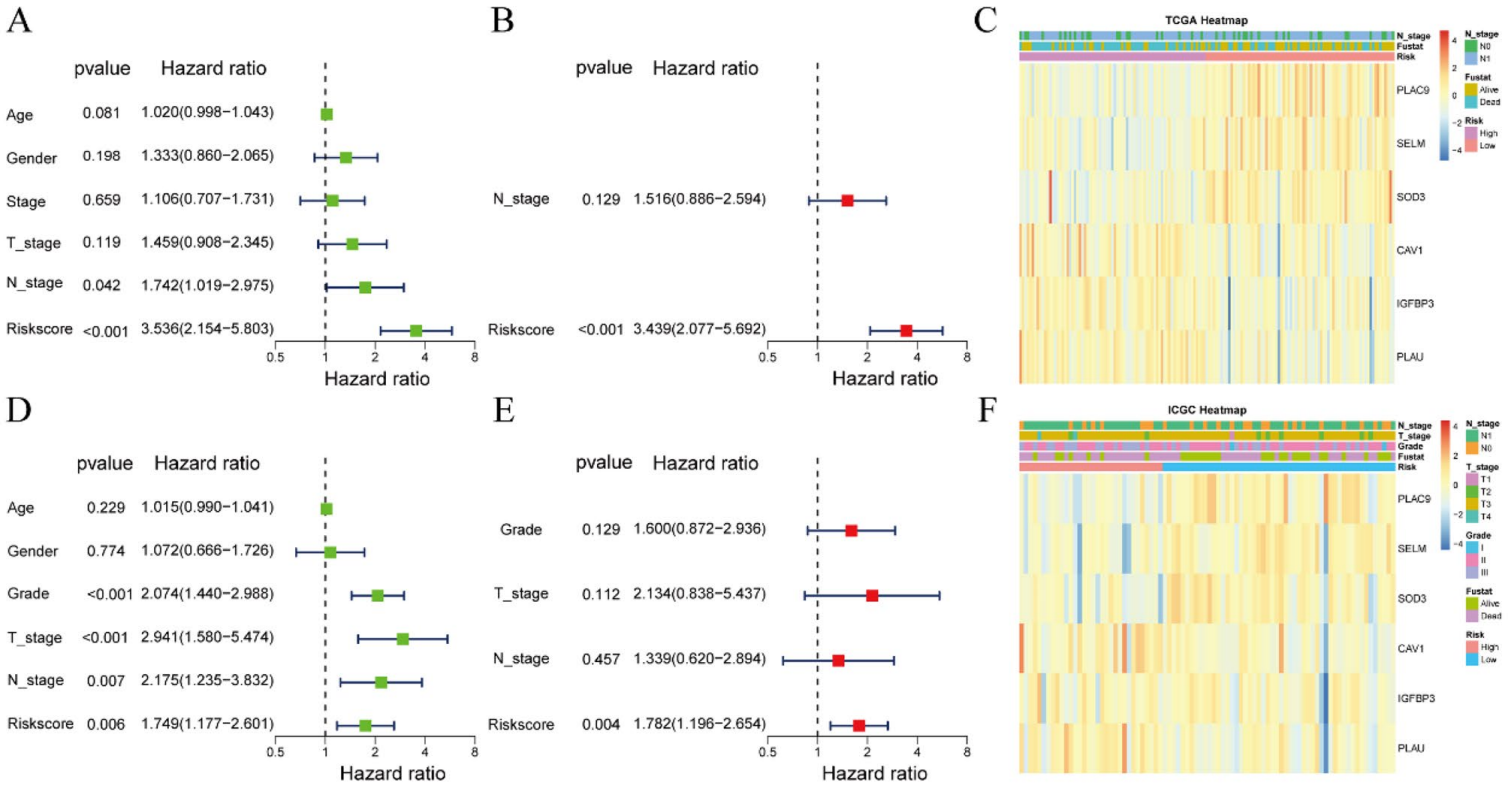
Prognostic value of clinical features and risk scores in patients with PAAD. (**A**) Univariate and (**B**) multivariate Cox regression analysis of factors associated with OS in PAAD patients in the TCGA cohort. (**C**) Heatmap showing the expression of the six prognostic genes and the distribution of clinical features and risk groups in the TCGA cohort. (**D**) Univariate and (**E**) multivariate Cox regression analysis of factors associated with OS in PAAD patients in the ICGC cohort. (**F**) Heatmap showing the expression of the six prognostic genes and the distribution of clinical features and risk groups in the ICGC cohort.

Next, we performed univariate and multivariate Cox regression analysis to verify the independently prognostic value of clinical information and risk score. Univariate analysis of patients in the TCGA cohort showed that N stage and risk score were potential prognostic factors (Fig. 4A), with multivariate Cox regression analysis showing that risk score (hazard ratio [HR] 3.439, 95% confidence interval [CI] 2.077–5.692, P < 0.001) was independently prognostic factor for survival in patients with PAAD (Fig. 4B). A heatmap of the expression of the six prognostic genes, the distribution of clinical features and risk groups, and the survival status of PAAD patients in the TCGA cohort showed that the percentage of living patients was higher and clinical grade lower in the low-risk than in the high-risk group (Fig. 4C). Univariate Cox regression analysis of risk score and clinical features, including age, gender, tumor grade, T stage, and N stage, of PAAD patients in the ICGC cohort showed that tumor grade, T stage, N stage, and risk score were potential prognostic factors (Fig. 4D), with multivariate Cox regression analysis showing that risk score (HR 1.782, 95% CI 1.196–2.654, P = 0.004) has independent prognostic value for survival in patients with PAAD (Fig. 4E). Similar to findings in the TCGA cohort, a heatmap of the expression of the six prognostic genes, the distribution of clinical features and risk groups, and the survival status of PAAD patients in the ICGC cohort showed that the percentage of living patients was higher and clinical grade lower in the low-risk than in the high-risk group (Fig. 4F).

### Functional enrichment analyses based on prognostic signatures

**Figure 5:**
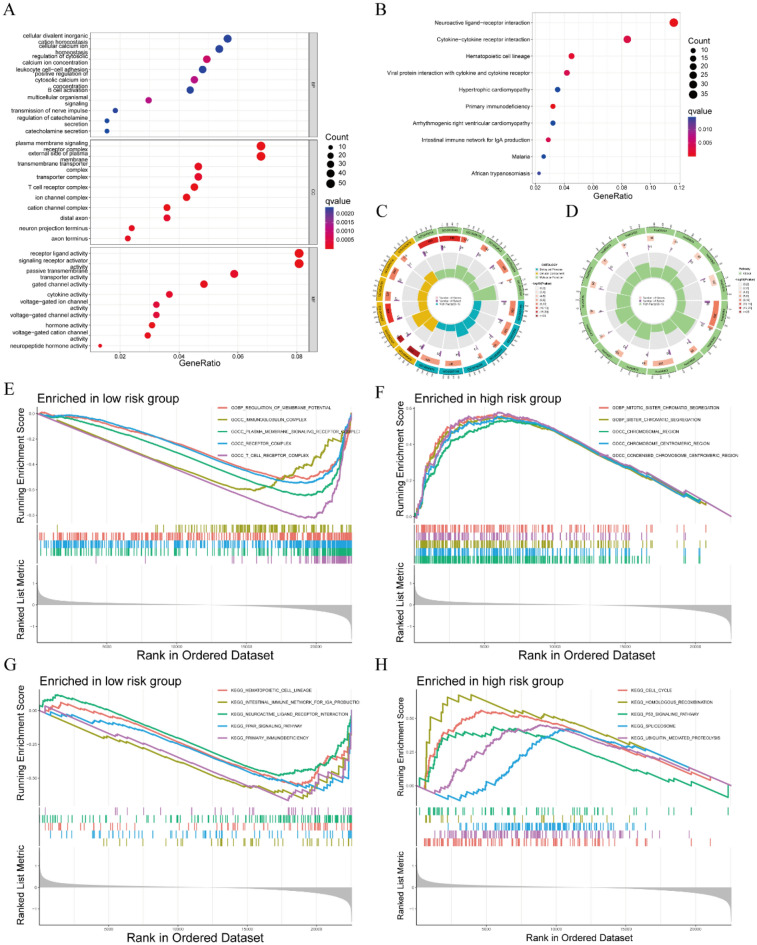
Functional enrichment analyses comparing the low-risk and high-risk subgroups. (**A**) GO enrichment analyses, including biological processes (BP), cellular components (CC), and molecular function (MF), of DEGs in the low-risk and high-risk groups. (**B**) KEGG enrichment analysis of the DEGs in the low-risk and high-risk groups. (**C,D**) Circle plots of the results of (**C**) GO and (**D**) KEGG enrichment analyses. (**E,F**) The top five enriched biological functions in the (**E**) low-risk and (**F**) high-risk groups. (**G,H**) The top five enriched pathways in the (**G**) low-risk and (**H**) high-risk groups.

The 1707 DEGs with |Log2FC| > 1 and P < 0.05 between low-risk group and high-risk group were identified using the “DESeq2” package to investigate the differences in biological function and pathway of two subgroups. These DEGs were subjected to GO analysis, KEGG analysis, and GSEA using the “clusterProfiler” package. The biological functions of most of these DEGs mainly focused on three cellular functions (Fig. 5A). The first was calcium ion-related functions, such as the regulation of cytosolic calcium ion concentration, cellular calcium ion homeostasis, ion channel complexes, and voltage-gated ion channel activity. The second was neurohormone-related functions, including the regulation of catecholamine secretion, the transmission of nerve impulses, catecholamine secretion, transmembrane transporter complex, hormone activity, and neuropeptide hormone activity. The third was immune-related functions, such as leukocyte cell–cell adhesion, B cell activation, T cell receptor complex, cytokine activity, and receptor ligand activity (Fig. 5A). DEGs associated with immune-related pathways were most highly enriched, including DEGs associated with neuroactive ligand-receptor interactions, primary immunodeficiency, cytokine-cytokine receptor interactions, the intestinal immune network for IgA production, and viral protein interactions with cytokines and cytokine receptors^22–24^ (Fig. 5B). Circle plots provided more detailed information on the results of GO and KEGG analyses, including p values and numbers of genes (Fig. 5C,D).

GSEA was also used to investigate the differences between the low- and high-risk groups. Biological functions enriched in the low-risk group included T cell receptor complexes, immunoglobulin complexes, and receptor complexes (Fig. 5E), whereas biological functions enriched in the high-risk group included tumor proliferation-related functions, such as sister chromatid segregation, condensed chromosomal center, and mitotic sister chromosome segregation (Fig. 5F). Other pathways enriched in low-risk group included the PPAR signaling pathway, primary immunodeficiency, and the gut immune network for IGA production, and so on (Fig. 5G), whereas other pathways enriched in high-risk group included tumor proliferation-related pathways, such as the cell cycle, homologous recombination and the p53 signaling pathway (Fig. 5H).

### Immune cells and correlation analysis based on risk score

**Figure 6:**
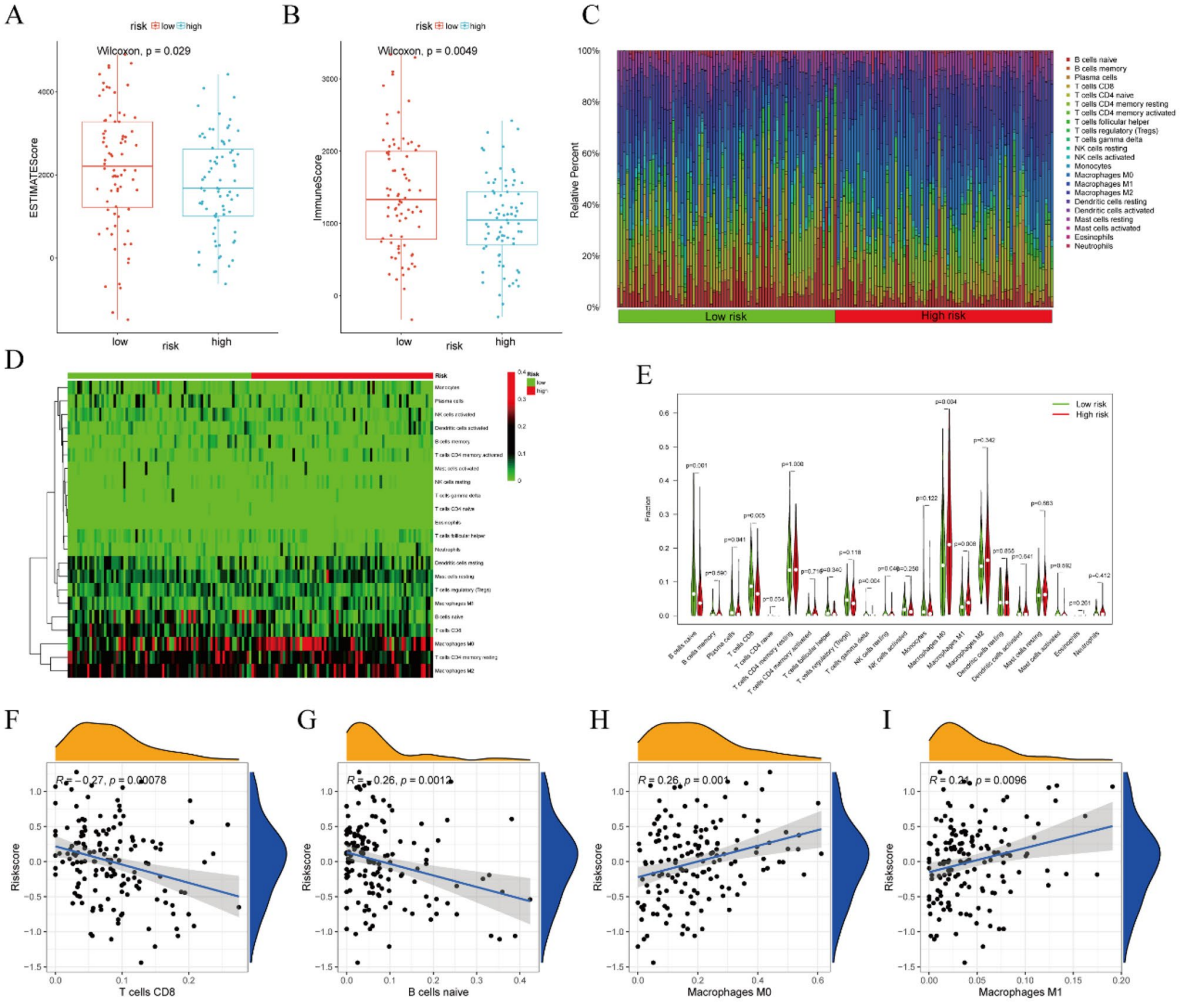
Immune cell infiltration and correlation analysis based on risk score. (**A**) Differences of ESTIMATE score between high-risk and low-risk groups. (**B**) Differences of Immune score between high-risk and low-risk groups. (**C**) Infiltration of 22 types of immune cells into the tumor microenvironment (TME) of PAAD patients in the high- and low-risk groups. (**D**) Heatmap showing the degree of infiltration of immune cells in the TME of low- and high-risk groups. (**E**) Differences in immune cells fractions between the low- and high-risk groups. (**F**) Correlation between CD8 T cell infiltration and risk score. (**G**) Correlation between naïve B cell infiltration and risk score. (**H**) Correlation between M0 macrophages and risk score. (**I**) Correlation between M1 macrophages infiltration and risk score.

These enrichment analyses indicated that several immune-associated pathways and processes were enriched in both the low-risk and high-risk groups. By using the ESTIMATE algorithm, we found that the immune score and ESTIMATE score were higher in the low-risk group than those in the high-risk group, which indicated there was the higher level of immune cell infiltration in the low-risk group (Fig. 6A,B). The infiltration into the TME of 22 types of immune cells was evaluated in 151 PAAD samples using the “CIBERSORT” package (Fig. 6C). A heatmap showed that M0 macrophages, M2 macrophages, CD8 T cells, and resting memory CD4 T cells accounted for large proportions of immune cells infiltration all PAAD samples (Fig. 6D). In addition, the levels of infiltration of CD8 T cells, naïve B cells, plasma cells, and resting NK cells were higher in the low-risk than in the high-risk group. Conversely, the levels of infiltration of M0 macrophages and M1 macrophages were lower in the low-risk than in the high-risk group (P < 0.05, Fig. 6E).

Spearman rank correlation analysis evaluating the correlation between the proportions of immune cells and risk scores showed that the proportions of CD8 T cells (P = 0.00078, R =  − 0.27) and naïve B cells (P = 0.0012, R =  − 0.26) were negatively correlated with risk scores, indicating that lower scores were associated with higher proportions of CD8 T cells and naïve B cells (Fig. 6F,G). On the contrary, the proportions of M0 Macrophages (P = 0.001, R = 0.26) and M1 Macrophages (P = 0.0096, R = 0.21) were positively correlated with risk scores, indicating that lower scores were associated with lower proportions of M0 Macrophages and M1 Macrophages (Fig. 6H,I).

### Somatic mutations in the low- and high-risk groups

**Figure 7:**
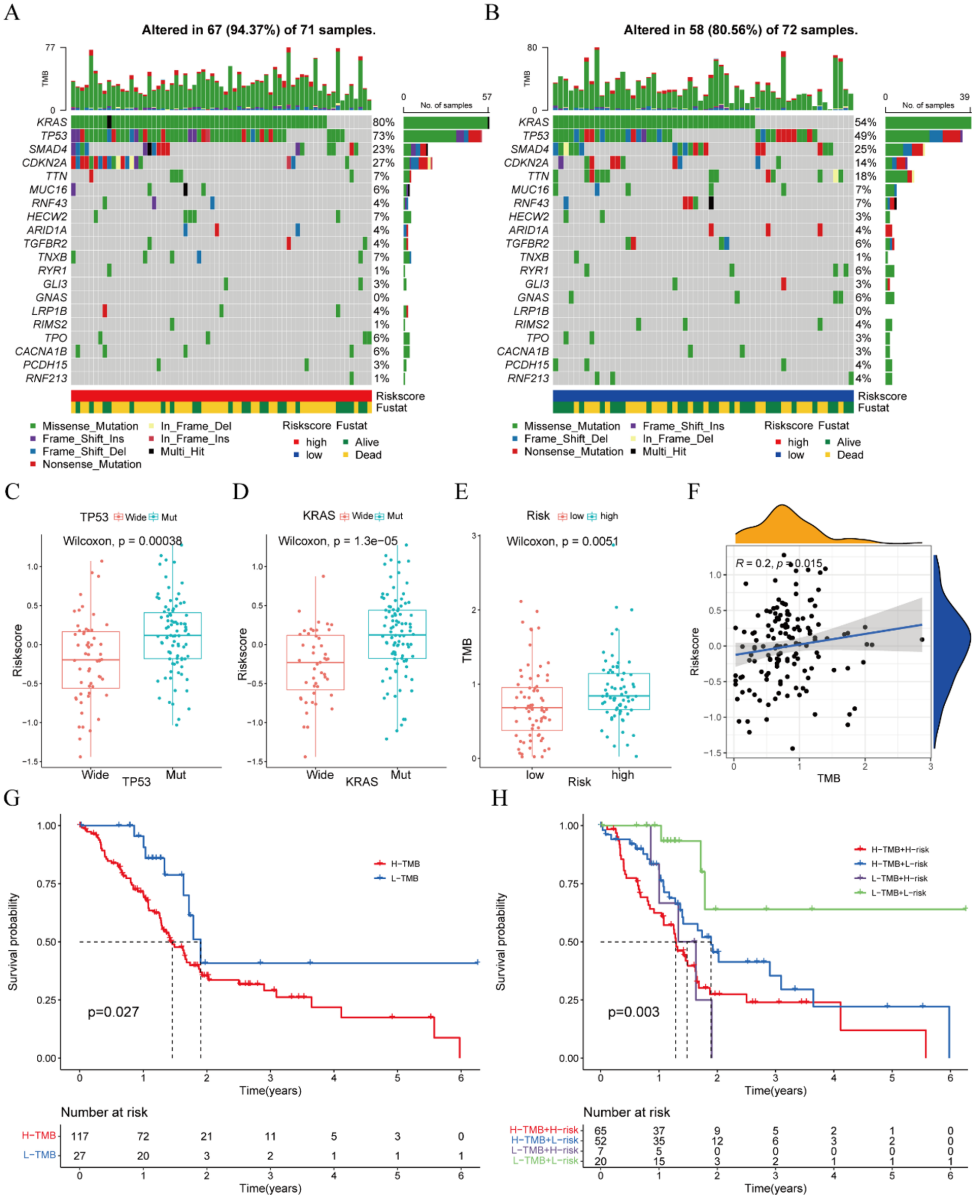
Somatic mutation analysis of samples in the low- and high-risk groups. (**A,B**) Waterfall plots of somatic mutations in the (**A**) high-risk and (**B**) low-risk groups. (**C**) Risk scores in patients with mutant and wild-type *TP53*. (**D**) Risk scores in patients with mutant and wild type *KRAS*. (**E**) TMB in the low- and high-risk groups. (**F**) Correlations between TMB and risk scores. (**G**) Kaplan–Meier analysis of OS of PAAD patients with low TMB or high TMB. (**H**) Survival analysis combining the risk and TMB subgroups.

The total mutation burden (TMB) and the number of genetic mutations in 143 PAAD patients with somatic mutation data were analyzed using the “Maftools” package. The five genes with the highest mutation frequencies in both the high- and low-risk groups were found to be *KRAS*, *TP53*, *SMAD4*, *CDKN2A*, and *TTN*, with missense mutations being the most common and frequent variant classification in both groups (Fig. 7A,B). Somatic mutations were observed in 67 (94.37%) of 71 samples in the high-risk group, compared with 58 (80.56%) of 72 samples in the low-risk group. Moreover, risk scores were significantly higher in patients with mutant than wild-type *KRAS* and *TP53* (Fig. 7C,D). The TMB was significantly higher in the high-risk than in the low-risk group, as shown by the Wilcoxon test, with TMB value and risk score being positively correlated (Fig. 7E,F). Next, we investigated the association between TMB, risk score, and OS of PAAD patients. Notably, patients with low TMB exhibited significantly longer survival times compared to those with high TMB (Fig. 7G). Survival analysis integrating the risk and TMB subgroups unveiled that patients in the low-risk group with low TMB demonstrated the most favorable prognosis, followed by those in the low-risk group with high TMB. Conversely, patients in the high-risk group, regardless of TMB status, exhibited the poorest prognosis (Fig. 7H).

### Drug sensitivity analysis based on the risk model

**Figure 8:**
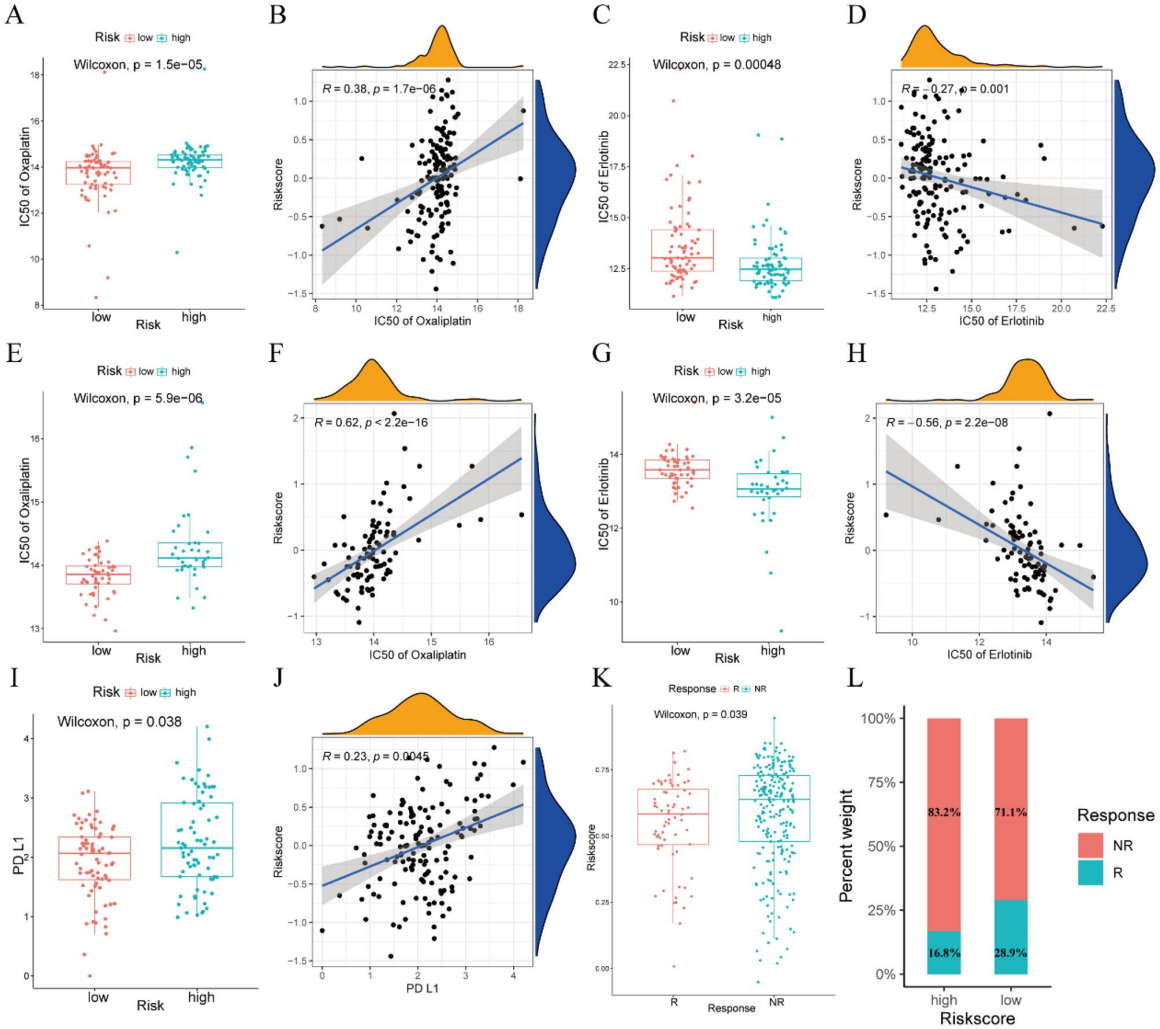
IC50s of oxaliplatin and erlotinib in PAAD patients with low and high-risk scores and correlations between these IC50s and risk score. (**A,B**) IC50s of oxaliplatin and correlation between IC50s and risk scores in the TCGA cohort. (**C,D**) IC50s of erlotinib and correlation between IC50s and risk scores in the TCGA cohort. (**E,F**) IC50s of oxaliplatin and correlation between IC50s and risk scores in the ICGC cohort. (**G,H**) IC50s of erlotinib and correlation between IC50s and risk score in the ICGC cohort. (**I**) Expression of PD-L1 in the low- and high-risk groups in the TCGA cohort. (**J**) Correlation between expression of PD-L1 and risk scores in the TCGA cohort. (**K**) The risk scores in groups with different anti-PD-L1 treatment response status in the IMvigor210 cohort. *NR:* progressive disease (PD)/stable disease (SD), *R:* complete response (CR)/partial response (PR). (**L**) The objective response rate in the low-risk and high-risk group.

The drug sensitivity of tumors in the low-risk and high-risk groups was assessed by calculating the IC50 of drugs most frequently used to treat PAAD, such as gemcitabine, paclitaxel, oxaliplatin, olaparib, fluorouracil, and erlotinib, using the “oncoPrediect” package. The IC50s of these chemotherapy drugs were calculated in the 151 patients in the TCGA cohort and the 90 patients in the ICGC cohort. Evaluation of the TCGA cohort showed that the IC50 of oxaliplatin was significantly lower in the low-risk than in the high-risk group, with the IC50 of oxaliplatin and the calculated risk score being positively correlated (R = 0.38, P < 0.001) (Fig. 8A,B). In contrast, the IC50 of erlotinib was significantly higher in the low-risk than in the high-risk group, with the IC50 of erlotinib and the risk score being negatively correlated (R =  − 0.27, P = 0.001) in the TCGA cohort (Fig. 8C,D). Similar results were observed in the ICGC cohort, with the IC50 of oxaliplatin being significantly lower in the low-risk than in the high-risk group and a significant positive correlation between the IC50 of oxaliplatin and risk score (R = 0.62, P < 0.001; Fig. 8E,F). Moreover, the IC50 of erlotinib was significantly higher in the low-risk than in the high-risk group, with the IC50 of erlotinib showing a significant negative correlation with risk score (R =  − 0.56, P < 0.001; Fig. 8G,H). In addition, we explored the relationship between PD-L1 expression and risk score in TCGA cohort. The expression of PD-L1 was significantly higher in the high-risk than in the low-risk group (P = 0.038), with the expression of PD-L1 and the risk score being positively correlated (R = 0.23, P = 0.0045) in the TCGA cohort (Fig. 8I,J). Lastly, the risk scores of 298 patients who received anti-PD-L1 treatment were calculated to further explore the power of this signature in predicting the immunotherapy response. There are lower risk scores in the objective response group to anti-PD-L1 treatment than in the non-response group (P = 0.039, Fig. 8K). The objective response rate in the low-risk group was significantly higher than in the high-risk group (Fig. 8L), which indicate that patients with a low-risk score are more likely to benefit from immunotherapy.

### Consensus clustering analysis based on the risk model

**Figure 9:**
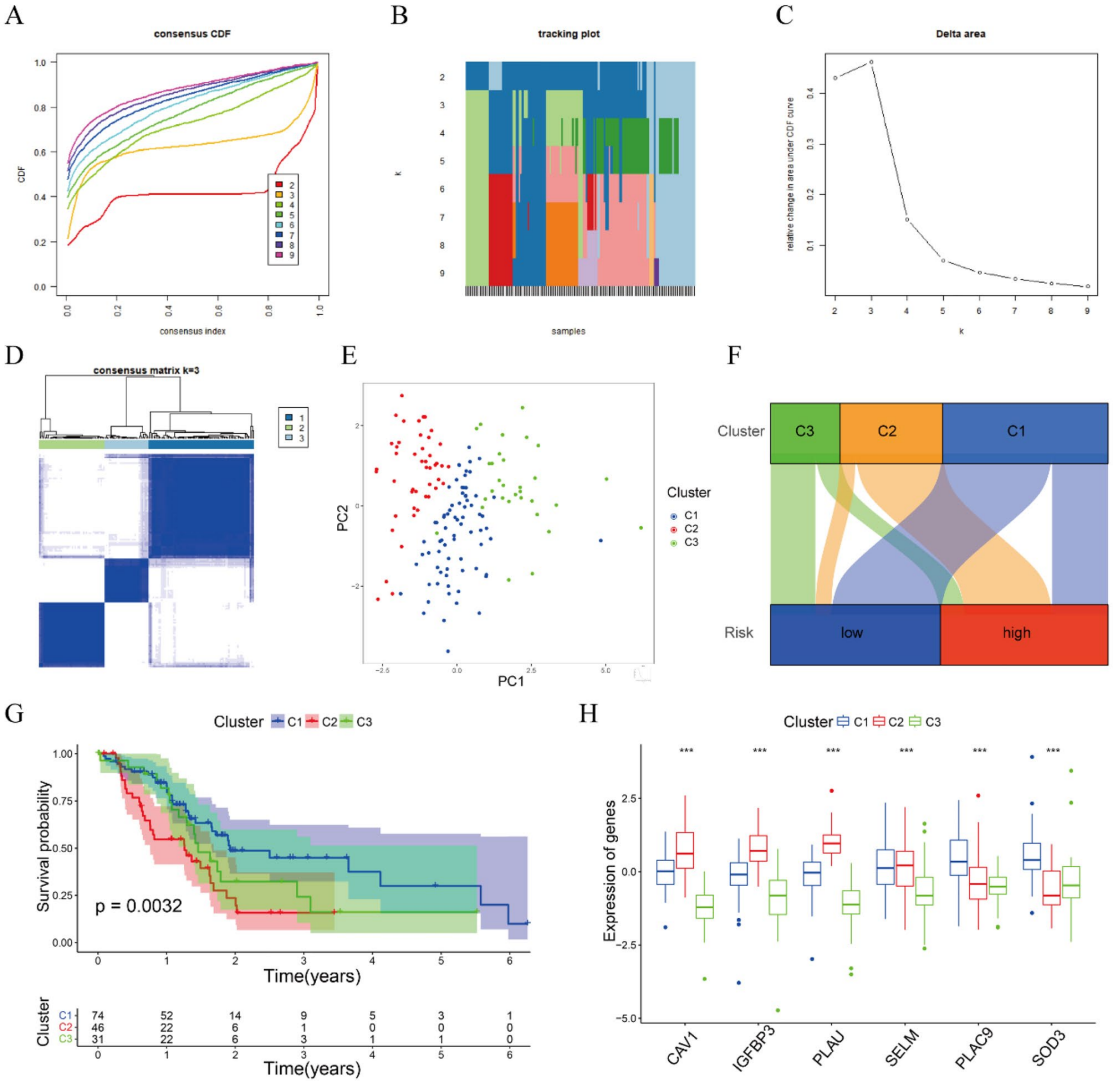
Consensus clustering analysis of the six genes included in the risk model for PAAD. (**A**) Cumulative distribution functions (CDF) of consensus clusters for k = 2–9. (**B**) Tracking plot for k = 2–9. (**C**) Relative changes in CDF delta areas at k = 2–9. (**D**) The consensus matrix for k = 3. (**E**) PCA plot showing that the 151 patients formed three clusters. (**F**) Alluvia diagrams of the three clusters and two risk groups. (**G**) Kaplan–Meier analysis of OS of PAAD in the three clusters, with comparisons by log-rank tests. (**H**) Differential expression of the six genes among the three clusters.

The impact of the six genes in risk model on survival outcomes in 151 PAAD patients was further investigated by unsupervised consensus clustering analysis using the “ConsensusClusterPlus” package in R. The optimal K = 3 was selected from k = 2 to 9 based on the lowest intergroup correlations and the highest intragroup correlations, and PAAD patients were divided into three clusters (C1, C2, and C3) (Fig. 9A–D). PCA clearly showed that these samples formed three clusters (Fig. 9E). The relationship between the three clusters and the two risk groups was explored via an alluvia diagram. Most patients in C2 were found to belong to the high-risk group, whereas most patients in C1 and C3 belonged to the low-risk group (Fig. 9F). Kaplan–Meier analysis showed that OS was significantly longer in C1 and C3 than in C2 (P = 0.0032; Fig. 9G). Investigation of the expression of the six genes in these three clusters showed that the levels of expression of *CAV1*, *IGFBP3*, and *PLAU* were higher in C2 than in C1 and C3 and the levels of *PLAC9* and *SOD3* were lower in C2 than in C1 (Fig. 9H). These findings indicated that *CAV1*, *IGFBP3*, and *PLAU* could be considered oncogenes and *PLAC9* and *SOD3* were associated with reduced risk.

### High expressions of CAV1 and SOD3 in CAFs

**Figure 10:**
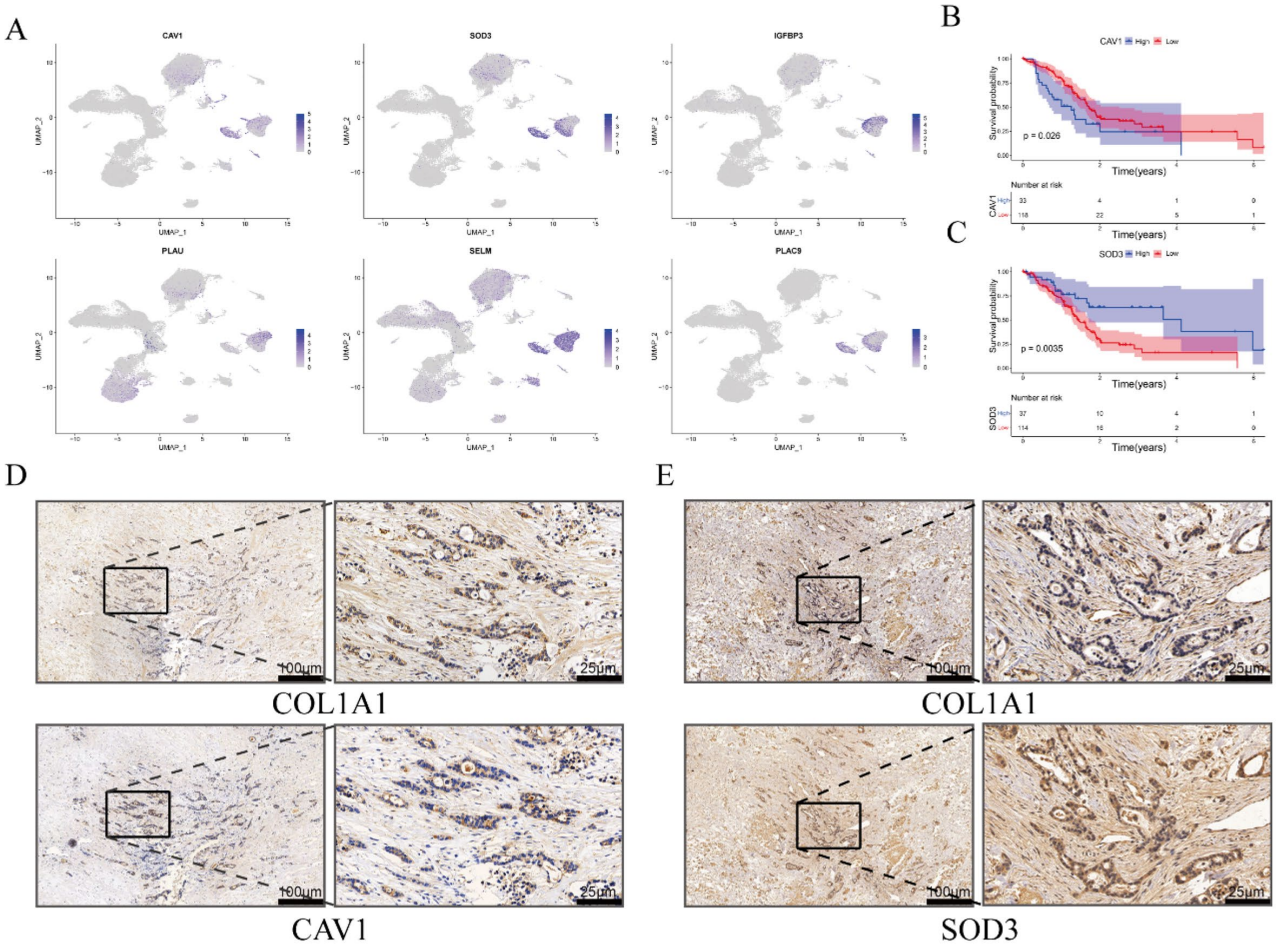
The expressions of CAV1 and SOD3 in PAAD tissue. (**A**) The feature plot of six genes in PAAD. (**B**) The OS of PAAD patients in the high expression group and low expression group of CAV1 from Kaplan–Meier analysis. (**C**) The OS of PAAD patients in the high expression group and low expression group of SOD3 from Kaplan–Meier analysis. (**D**) The expressions of CAV1 and COL1A1 in PAAD tissue*.* (**E**) The expressions of SOD3 and COL1A1 in PAAD tissue.

We explored the expressions of six genes for constructing prognostic signature in each cell cluster through feature plot, which shown all six genes were highly expressed in CAFs in PAAD (Fig. 10A). Then, the prognostic values of these genes were estimated via Kaplan–Meier analysis, and the best cutoff value of Kaplan–Meier analysis was obtained from the “survival” R package. We found the survival probability in the high-expression group of CAV1 were remarkably lower than that in the low-expression group of CAV1 (Fig. 10B) and the survival probability in the high-expression group of SOD3 were remarkably higher than that in the low-expression group of SOD3 (Fig. 10C). Then, we further performed immunohistochemical staining to explore the expressions of CAV1 and SOD3 in PAAD tissue. We found that CAV1 and SOD3 were highly expressed in fibroblasts characterized by the elevated expression of COL1A1 in PAAD (Fig. 10D,E). Meanwhile, we verified the high expression of SELM, IGFBP3, PLAC9, and PLAU at the mRNA level in fibroblasts of the pancreas through the Human Protein Atlas website (https://www.proteinatlas.org/). By combining the immunohistochemical staining results for SOD3 and CAV1 with the mRNA expression levels of SELM, IGFBP3, PLAC9, and PLAU, we aimed to provide a comprehensive understanding of the molecular characteristics and potential therapeutic targets associated with the fibroblast component in PAAD.

## Discussion

CAFs are the main constituent cells of the TME in patients with PAAD. CAFs interact with almost all other cells in the TME, regulating tumor progression and metastasis. The cytokines, exosomes, growth factors, chemokines, and other effector molecules secreted by CAFs are key factors in their interactions with immune cells that infiltrate tumors^15^. The present study describes the screening of 189 CAF marker genes by single cell sequencing, and the construction of a six-gene (*CAV1*, *IGFBP3*, *PLAC9*, *PLAU*, *SELM*, and *SOD3*) prognostic signature to evaluate the influence of CAF marker genes on the prognosis of patients with PAAD.

CAV1 is a scaffolding protein that can promote the formation of morphologically identifiable caveolae and regulate signal transduction molecules^25,26^. Several previous studies have shown that CAV1 was a significantly prognostic marker of PAAD and that its level of expression correlated significantly with the levels of p53, Ki-67, and CA19-9^27–30^. The molecule circFARP1 was reported to bind directly to CAV1, inhibiting the degradation of the latter and enhancing gemcitabine resistance of PAAD by increasing the secretion of leukemia inhibitory factor^31^. IGFBP-3 and its receptor IGFBP-3R have also been associated with chemoresistance and poor prognosis in patients with PAAD^32,33^. Overexpression of PLAC9 was shown to reduce lung cancer cell proliferation and increase their migration and invasion in vitro^34^. PLAU has been regarded as an immune-related gene and has been associated with OS in patients with PAAD^35–38^. Moreover, PLAU, as a prognostic marker, was found to promote CAFs conversion and the proliferation and migration of esophageal squamous cell carcinoma via the uPAR/Akt/NF-κB/IL8 pathway^39^. Loss of SOD3 was shown to promote the invasion and migration of PAAD by increasing reactions of superoxide with nitric oxide^40^. These findings indicate that the six genes included in the risk model can predict the prognosis of patients with PAAD patients. In addition, immunohistochemical staining showed the high expression of CAV1 and SOD3 on CAF, which verified the reliability of prognostic signature.

Evaluation of the infiltration of 22 types of immune cells into the TME of low-risk and high-risk PAAD showed that the levels of infiltration of CD8 T cells, naïve B cells, plasma cells, and resting NK cells were higher in the low-risk group, whereas the levels of infiltration of M0 and M1 macrophages were higher in the high-risk group. CD8 T cells are the main effector cells that attack tumor cells in the TME. PAAD cells are recognized by CD8 T cells as foreign bodies in a major histocompatibility complex class I-restricted manner^41^. These CD8 T cells are subsequently activated, attacking tumor cells with tumor-associated antigens on their surfaces^42^. Tumors with high levels of CD8 T cell infiltration in the TME are regarded as immunogenically hot tumors, which respond better to immune checkpoint inhibitors^43^. B cells are vital players in the core immune network, restraining recurrence and tumor progression at late stage, thereby prolonging patient survival^44^.

M1 macrophages, which specifically overexpress iNOS, HLA-DR, CD80, CD86, and other molecules, can improve the survival of patients by promoting Th1 anti-tumorigenic or immunostimulatory responses^45^. These findings would suggest that OS may be longer for patients in the high-risk group with higher infiltration of M1 macrophages than for patients in the low-risk group, results contrary to the prediction of the present risk model. However, large numbers of M2 macrophages are present in the TME of both groups. M2 macrophages promote a Th2, pro-tumorigenic or immunosuppressive response, resulting in a generally immunosuppressive environment^46,47^.

The present study also compared somatic mutations and drug sensitivity in the low- and high-risk groups, as well as the correlation of these factors with risk scores. The mutation rates of *KRAS* and *TP53* were found to be significantly lower in the low-risk than in the high-risk group. The *KRAS* and *TP53* genes are frequently mutated in PAAD, with both greatly affecting various aspects of the TME^48^. KRAS mutations activate the critical GTP/GDP GTPase exchange protein, a molecular switch that activates various intracellular signaling pathways that regulate the proliferation and metastasis of PAAD, thereby affecting patient survival^49^. Moreover, KRAS mutations can result in the overexpression of the immune check point regulator programmed death-ligand 1 (PD-L1) to accelerate the formation of immunosuppressive TME^50,51^. TP53 mutations can also alter the TME and promote pro-tumorigenic associated inflammation, accelerating PAAD cell proliferation and metastasis^52^. Specifically, TP53 mutations can promote NF-κB activity to induce the expression of pro-inflammatory cytokines such as IL-6 and TNF-α, which promote the metastasis of PAAD^53,54^. What’s more, our research proved that patients harboring low TMB or high TMB in low-risk group had better prognosis than those having low TMB or high TMB in high-risk group. So we believed that both TMB and signature were important prognostic indicators for PAAD patients, and this signature had more accurate predictive ability than pure KRAS or TP53 mutation in some patients.

The FOLFIRINOX regimen, consisting of a combination of oxaliplatin, irinotecan, fluorouracil, and leucovorin, as well as combinations of gemcitabine with conventional drugs, are the most commonly used chemotherapy regimens for the treatment of PAAD^55–57^. The present study found that the patients in the low-risk group were more sensitive to oxaliplatin, indicating FOLFIRINOX would prolong survival for patients in this group. In addition, the situation that the objective response rate in the low-risk group with high PD-L1 expression was significantly higher than in the high-risk group with low PD-L1 expression caught our attention. The efficacy of PD-L1 therapy is influenced by the expression of PD1 and PD-L1, the CD8 T infiltration cell, and other factors within the complex TME. In PAAD, the dense fibrous stroma, predominantly composed of collagen, hyaluronic acid (HA), and fibronectin, plays a crucial role. Our findings indicated that the expression levels of hyaluronan synthase 3 (HAS3) and vitamin D receptor (VDR) were higher in the high-risk group compared to the low-risk group (Fig. S1). HAS3 is responsible for promoting hyaluronic acid synthesis, while elevated expression of VDR activates pancreatic stellate cells (PSCs) and contributes to the extensive stromal reaction observed in PAAD^58^. We hypothesized that the tighter extracellular matrix in the high-risk group may lead to a diminished response to PD-L1 therapy. Additionally, we observed higher expression of NT5E, also known as CD73, in the high-risk group compared to the low-risk group (Fig. S1). Notably, increased CD73 expression has been shown to significantly impede the efficacy of PD-L1 therapy^59^. These findings supported that the low-risk group may exhibit improved responsiveness to PD-L1 therapy.

In conclusion, the present study described the construction and validation of a six-gene prognostic signature based on CAF marker genes that could predict prognosis, TMB, and drug sensitivity in patients with PAAD. The genes included in this signature may serve as potential therapeutic targets and prognostic biomarkers to improve the survival rate of patients with PAAD.

## Materials and methods

### Data collection

Single-cell RNA-sequencing (RNA-seq) data on tumor samples from 30 patients with PAAD were obtained from the GSE154778 (n = 10), GSE155698 (n = 15), and GSE156405 (n = 5) datasets in the Gene Expression Omnibus (GEO) database (https://www.ncbi.nlm.nih.gov/geo/) and used to identify CAF marker genes in PAAD. In addition, RNA-seq expression data and corresponding clinical information for 178 PAAD samples were downloaded from The Cancer Genome Atlas (TCGA) database (https://portal.gdc.cancer.gov/). Based on merged sample quality annotations (https://gdc.cancer.gov/about-data/publications/pancanatlas), 151 samples were enrolled in this study. Furthermore, somatic mutation data on 143 PAAD samples were downloaded from TCGA database, and RNA-seq expression data and related clinical information on 90 PAAD samples were downloaded from the International Cancer Genome Consortium (ICGC) database (https://dcc.icgc.org/). The RNA-seq data and matched clinical data of 298 patients with urothelial carcinoma who received anti-PD-L1 treatment were collected from the IMvigor210 cohort (http://research-pub.gene.com/IMvigor210CoreBiologies/) to explore the value of CAF-associated signature in speculating on the immunotherapy response. All RNA-seq data were normalized as transcripts per million (TPM) and log2-transformed for subsequent analysis.

### Identification of CAF marker genes by scRNA-seq analysis

Based on standard workflow for Seurat single-cell analysis, a Seurat object was created for each of the 30 PAAD datasets^60^. These 30 Seurat objects, which included 69,371 cells, were merged into a single Seurat object. To obtain high-quality scRNA-seq data, cells with < 100 measured genes, cells with > 20% mitochondrial contamination, and cells with > 6000 measured genes were removed, with the remaining 57,486 high-quality cells selected for subsequent analysis. The merged object was normalized via the “NormalizeData” function and the batch effect of the 30 samples was corrected using the “Harmony” package. Twenty harmony dimensions were evaluated, and the top two uniform manifold approximation and projection (UMAP) dimensions were visualized at a clustering resolution of 0.2. The 20 cell clusters were annotated as 10 cell clusters each based on previously described cell markers^20^ and on CellMarker (http://xteam.xbio.top/CellMarker/index.jsp). The cell markers included B cells (*CD79A*, *CD19*, *MS4A1*), acinar cells (*PRSS1*), T cells (*CD3D, IL17R*), NKT cells (*CD3D*, *IL17R*, *FCGR3A*, *NKG7*, *GNLY*), epithelial cells (*EPCAM, CDH1, KRT8*), fibroblasts (*ACTA2*, *COL1A1*), myeloid cells (*CD14*, *LYZ*, *FCGR3A*), endothelial cells (*PECAM1*, *CDH5*), plasma cells (*IGJ*, *CD79A*), and mast cells (*CPA3*, *TPSAB3*). The differentially expressed genes (DEGs) in each cluster were identified using the “FindAllMarkers” function of the “Seurat” package. The 189 DEGs of fibroblast clusters with |Log2FC| > 1 and P-value < 0.05, adjusted using the BH method, were considered CAF marker genes and their protein–protein interaction (PPI) network was obtained from Search Tool for the Retrieval of Interacting Genes (STRING) (https://www.string-db.org/, version 11.5) (Table S1).

### Construction and validation of the prognostic signature for CAF marker genes

Univariate Cox proportional hazards regression analysis of PAAD patients in the TCGA cohort found that seven of the 189 DEGs, *CAV1*, *FXYD1*, *IGFBP3*, *PLAC9*, *PLAU*, *SELM*, and *SOD3*, had P values < 0.05; these seven DEGs were identified as associated with survival in patients with PAAD. Using the “glmnet” package, ten-fold cross-validation of the LASSO-penalized Cox regression analysis was performed to select the most suitable DEGs. The six genes identified, *CAV1*, *IGFBP3*, *PLAC9*, *PLAU*, *SELM*, and *SOD3*, and their coefficients were used to construct a prognostic signature, with a penalty parameter (λ) selected according to minimum criteria. The risk score for each patient was calculated based on regression coefficients derived from the LASSO-Cox regression model multiplied by the level of expression of each gene, using the equation: Risk score = ${\sum }_{i}^{6}Xi\times Yi$, where X represents the coefficients and Y represents the level of gene expression. To remove the batch effect between the TCGA and ICGC cohorts, all gene expression data were centralized and standardized using the “Scale” function. The median risk score of the 151 patients in the TCGA cohort was calculated, and these patients, as well as the 90 patients in the ICGC cohort, were separately divided into high- and low-risk groups based on the median risk score in the TCGA cohort. The performance of the prognostic signature was evaluated by Kaplan–Meier analysis of overall survival (OS) of patients in the low- and high-risk groups in each cohort, with comparisons by log-rank tests. In addition, receiver operating characteristic (ROC) curves for 1, 3, and 5 years OS were constructed, and the areas under the ROC curves (AUCs) calculated to verify the performance of the prognostic signature.

### Independent prognostic analysis of clinical features and risk score

The relationships among risk score and clinical features, including age, sex, and tumor stage, and OS of patients in the TCGA and ICGC cohorts were evaluated by univariate and multivariate Cox regression analyses. Independent prognostic factors were identified in the TCGA cohort and validated in the ICGC cohort.

### Gene set enrichment analysis and functional enrichment analysis based on the DEGs of the two subgroups

The 1707 DEGs between the low- and high-risk groups were identified using the “DESeq2” package, with |Log2FC| > 1 and P < 0.05 defined as statistically significant (Table S2). Using the “clusterProfiler” package, functional differences in the two groups were evaluated by Gene Ontology (GO) and Kyoto Encyclopedia of Genes and Genomes (KEGG) enrichment analyses. In addition, the “clusterProfiler” package was used to assess functional and pathway differences of the two groups at the gene set level, with the gene sets “c2.cp.kegg.symbols.gmt” and “c5.go.symbols.gmt” from GSEA (https://www.gsea-msigdb.org/gsea/msigdb) selected for reference. P < 0.05 in Functional Enrichment Analysis and GSEA was considered statistically significant.

### Evaluation of immune cells and correlation analysis

The relative proportions of 22 types of tumor-infiltrating immune cells in 151 PAAD samples were estimated using the “CIBERSORT” package, with differences of relative proportion in the two subgroups calculated using the “limma” package (Table S3). Additionally, the correlations between the proportions of immune cells and risk scores were evaluated using Spearman rank correlation analysis.

### Somatic mutation analysis and correlation analysis

Data on somatic mutations in tumors from 144 patients with PAAD were downloaded from TCGA. The total mutation burden (TMB) and the number of genetic mutations in each sample were calculated and illustrated using the “Maftools” package (Table S4). A patient with too high and outlier TMB was excluded from this analysis study. Differences in risk scores between patients having wild-type and mutant forms of frequently mutated genes were determined using the Wilcoxon rank-sum test. Relationships between risk scores and TMBs were evaluated using Spearman rank correlation analysis.

### Drug sensitivity and immunotherapy response analyses

Cancer Therapeutics Response Portal (CTRP) with information on drug sensitivity in cancer cells and molecular markers was used as training set. The ridge regression model was contracted based on CTRP gene expression profile and corresponding drug response information via the “oncoPredict” package. Then the half-maximal inhibitory concentrations (IC50s) of 545 drugs in patients with PAAD was predicted based on the sensitivity scores. Relationships between risk scores and IC50s were evaluated using Spearman rank correlation analysis. Additionally, 298 urothelial carcinoma patients who received anti-PD-L1 treatment from the IMvigor210 cohort were used for speculating the immunotherapy response of the signature.

### Immunohistochemical staining

The PAAD tissues were obtained from The Second Affiliated Hospital of Chongqing Medical University. Paraffin sections were placed in a 60 °C oven to melt the paraffin and immersed in xylene and ethanol at 100%, 95%, 80%, 75%, and 60% concentrations to elute the paraffin. The sections were immersed in boiling EDTA repair solution for 10 min and allowed to cool naturally. Then, the sections were incubated with 3% H_2_O_2_ at room temperature for 10 min to eliminate endogenous peroxidase activity. Then, the sections were incubated with 5% BSA blocking solution at 37 °C for 30 min. The sections were incubated with appropriately diluted CAV1 (afantibody, AF300083), SOD3 (afantibody, AF301460) and COL1A1 (abcam, ab138492) primary antibody at 4 °C overnight. The next day, the sections were incubated with secondary antibodies at room temperature for 60 min. After washing with PBS for 10 min three times, the tissues were stained with DAB and hematoxylin. Then, the sections were sequentially immersed in 60%, 75%, 80%, 95%, and 100% ethanol for dehydration. Finally, the sections were sealed with neutral gum and observed with a light microscope.

### Statistical analysis

All statistical analyses and plot were completed using R software (v4.2.1, https://cran.r-project.org/src/base/R-4/). OS in each group was calculated using the Kaplan–Meier method, with differences between groups evaluated using two-sided Log rank tests. Factors independently prognostic of OS, including the risk signature, were evaluated by univariate and multivariate Cox regression models, with correlations analyzed by Spearman’s test. P < 0.05 was defined as statistically significant.

### Ethics declarations

The protocols used in this research were evaluated and approved by the Ethics Committee of the First Affiliated Hospital of Chongqing Medical University (2020-358) and the written informed consent was obtained from all subjects. All methods of this study were carried out in accordance with the Declaration of Helsinki.

### Supplementary Information

Supplementary Figure S1.

Supplementary Table S1.

Supplementary Table S2.

Supplementary Table S3.

Supplementary Table S4.

## Data Availability

The TCGA-PAAD dataset for this study can be found in the TCGA database (https://portal.gdc.cancer.gov/); the GSE154778, GSE155698, and GSE156405 datasets used in this study can be downloaded from the GEO database (https://www.ncbi.nlm.nih.gov/geo/); the PACA-AU dataset used in this study can be found in the ICGC database (https://dcc.icgc.org/); the IMvigor210 cohort used in this study can be downloaded from http://research-pub.gene.com/IMvigor210CoreBiologies/.
