# Supplementary figures and images for: Cancer-associated fibroblast-related prognostic signature predicts prognosis and immunotherapy response in pancreatic adenocarcinoma based on single-cell and bulk RNA-sequencing
Source: Sci Rep. 2023 Sep 29;13:16408. doi: 10.1038/s41598-023-43495-y (PMC10541448; doi:10.1038/s41598-023-43495-y)

Figure S1


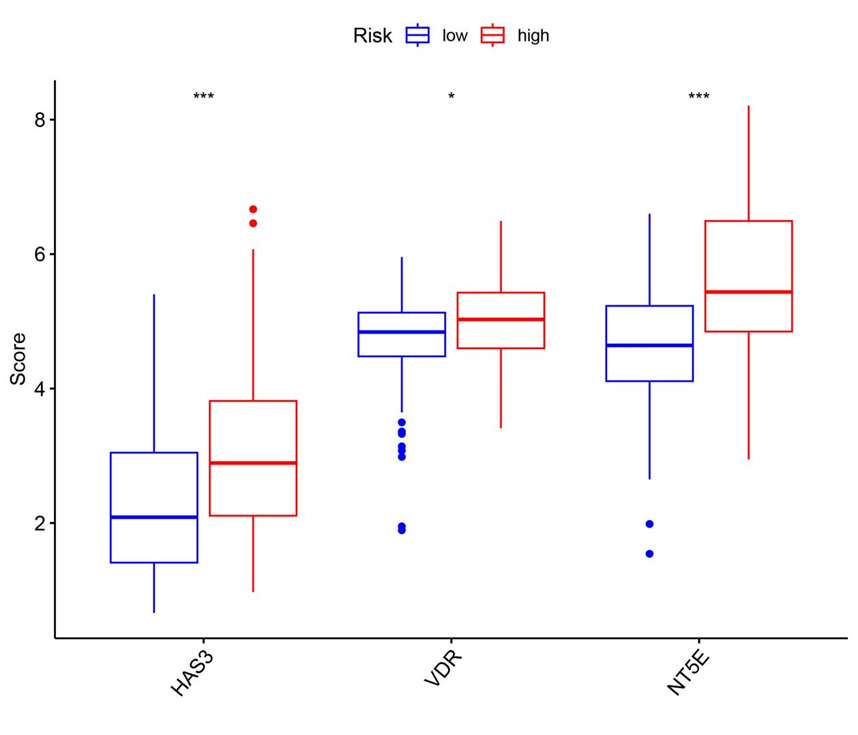


The expressions of the HAS3, VDR, and NT5E in the low risk and high risk group.

Supplement: Supplementary file 1 — Supplementary Figure S1. [file 41598_2023_43495_MOESM1_ESM.docx]
